# Impact of Temporal Resolution and Methods for Correction on Cardiac Magnetic Resonance Perfusion Quantification

**DOI:** 10.1002/jmri.28180

**Published:** 2022-03-26

**Authors:** Xenios Milidonis, Muhummad Sohaib Nazir, Amedeo Chiribiri

**Affiliations:** ^1^ School of Biomedical Engineering & Imaging Sciences King's College London London UK

**Keywords:** myocardial perfusion quantification, myocardial blood flow, temporal resolution, perfusion phantom, Monte Carlo simulations

## Abstract

**Background:**

Acquisition of magnetic resonance first‐pass perfusion images is synchronized to the patient's heart rate (HR) and governs the temporal resolution. This is inherently linked to the process of myocardial blood flow (MBF) quantification and impacts MBF accuracy but to an unclear extent.

**Purpose:**

To assess the impact of temporal resolution on quantitative perfusion and compare approaches for accounting for its variability.

**Study Type:**

Prospective phantom and retrospective clinical study.

**Population and Phantom:**

Simulations, a cardiac perfusion phantom, and 30 patients with (16, 53%) or without (14, 47%) coronary artery disease.

**Field Strength/Sequence:**

3.0 T/2D saturation recovery spoiled gradient echo sequence.

**Assessment:**

Dynamic perfusion data were simulated for a range of reference MBF (1 mL/g/min–5 mL/g/min) and HR (30 bpm–150 bpm). Perfusion imaging was performed in patients and a phantom for different temporal resolutions. MBF and myocardial perfusion reserve (MPR) were quantified without correction for temporal resolution or following correction by either MBF scaling based on the sampling interval or data interpolation prior to quantification. Simulated data were quantified using Fermi deconvolution, truncated singular value decomposition, and one‐compartment modeling, whereas phantom and clinical data were quantified using Fermi deconvolution alone.

**Statistical Tests:**

Shapiro–Wilk tests for normality, percentage error (PE) for measuring MBF accuracy in simulations, and one‐way repeated measures analysis of variance with Bonferroni correction to compare clinical MBF and MPR. Statistical significance set at *P* < 0.05.

**Results:**

For Fermi deconvolution and an example simulated 1 mL/g/min, the MBF PE without correction for temporal resolution was between 55.4% and −62.7% across 30–150 bpm. PE was between −22.2% and −6.8% following MBF scaling and between −14.2% and −14.2% following data interpolation across the same HR. An interpolated HR of 240 bpm reduced PE to ≤10%. Clinical rest and stress MBF and MPR were significantly different between analyses.

**Data Conclusion:**

Accurate perfusion quantification needs to account for the variability of temporal resolution, with data interpolation prior to quantification reducing MBF variability across different resolutions.

**Level of Evidence:**

3

**Technical Efficacy Stage:**

1

Quantitative myocardial magnetic resonance (MR) perfusion imaging is steadily being introduced into clinical routine for the assessment of coronary artery disease (CAD),^1^, ^2^, ^3^, ^4^ with recent guidelines recommending it for the assessment of acute chest pain.^5^ However, quantification pipelines are typically assessed against reference methods as a whole, neglecting any potential systematic errors caused by individual factors or parameters within the pipeline on perfusion measurements. One factor with direct impact on myocardial perfusion quantification is the temporal resolution of the sampled signals. Acquisition of the dynamic perfusion series is synchronized to the patient's cardiac cycle using prospective electrocardiogram (ECG) triggering. Consequently, the heart rate (HR) determines the sampling rate and the temporal resolution of the acquisition.

While myocardial blood flow (MBF) and myocardial perfusion reserve (MPR) are known to be correlated with resting HR due to changes in cardiac work,^6^ the impact of the associated sparsity in sampling rate during image acquisition on the mathematical derivation of MBF is largely unexplored. From the perspective of signal processing, sampling rate has a direct effect on deconvolution‐based perfusion quantification.^7^ Signals must be discretized before deconvolution is carried out under the assumption that they are uniformly sampled over time.^8^ It has been demonstrated that when deconvolving signals using a Fourier transform, the interval between consecutive time points affects the frequency resolution of the transform.^7^, ^9^ If uniformity in sampling rate is not enforced, the shape of the resulting impulse response function (IRF) and consequently the estimated MBF may vary. On the other hand, it is assumed that tracer‐kinetic models estimating MBF directly as a fitting parameter do not require sampling rate uniformity,^10^ but this may not be true for abnormally low or high rates.

A reasonable approach to account for the sparsity in sampling rate is interpolation of the signals to a fixed temporal resolution. The importance of interpolation for signal‐based flow measurement has been described as early as the 1960s,^11^, ^12^ and has been well‐studied in the quantification of cerebral perfusion with deconvolution techniques.^7^, ^13^, ^14^ In myocardial perfusion quantification, only a small fraction of studies have reported its use, while the associated improvement on MBF estimation remains unclear.^10^, ^15^, ^16^ Interpolation can be performed using linear,^10^, ^17^ piecewise cubic Hermite,^15^, ^18^ and piecewise cubic spline polynomials.^16^ Others have corrected MBF by scaling it by the inverse of the sampling interval, although how the sampling interval was estimated given the variable HR across perfusion scans was not described.^19^, ^20^

Thus, the aim of this study was to perform a comprehensive assessment of the impact of temporal resolution on myocardial perfusion quantification and determine the accuracy of aforementioned approaches for accounting for its variability.

## Materials and Methods

The impact of temporal resolution was assessed on data with three different levels of complexity: Monte Carlo simulations, phantom, and clinical data. Simulations were performed first and allowed determination of the most accurate approaches to be used for the analysis of phantom and clinical data. Simulations and analysis approaches were based on the principles of the indicator dilution theory and the central volume theorem described in detail in the Supporting Information. The clinical study was approved by the national ethics committee (15/NS/0030), and all patients gave written informed consent.

### Simulations

To represent real continuous contrast agent (CA) concentration curves yielding exact MBF values, highly dense reference arterial input functions (AIF) and myocardial tissue curves capturing the full CA clearance from arterial and myocardial sites were generated (simulated sampling frequency: 30 Hz, total duration: 20 minutes). Specifically, a single AIF simulating the traversal of an impulse CA injection through various organs before reaching the myocardium was produced as described previously.^20^, ^21^ The tissue curves were produced for five different physiological MBF values (1 mL/g/min, 2 mL/g/min, 3 mL/g/min, 4 mL/g/min, and 5 mL/g/min) and a fixed myocardial blood volume of 0.3 mL/g, so that realistic mean transit times (MTTs) were also obtained (18 seconds, 9 seconds, 6 seconds, 4.5 seconds, and 3.6 seconds, respectively).^22^ This was achieved by first using the Fermi function [equation (6), Supporting Information] to generate tissue residue functions with an area corresponding to the five MTT values [according to equation (2), Supporting Information]. Simulated tissue residue functions were then scaled to obtain IRF yielding the required MBF values. For the purpose of simulations, the myocardial density was assumed to be 1 g/mL. Finally, AIF and IRF were convolved to produce the simulated tissue curves [according to equation (1), Supporting Information].

**FIGURE 1:**
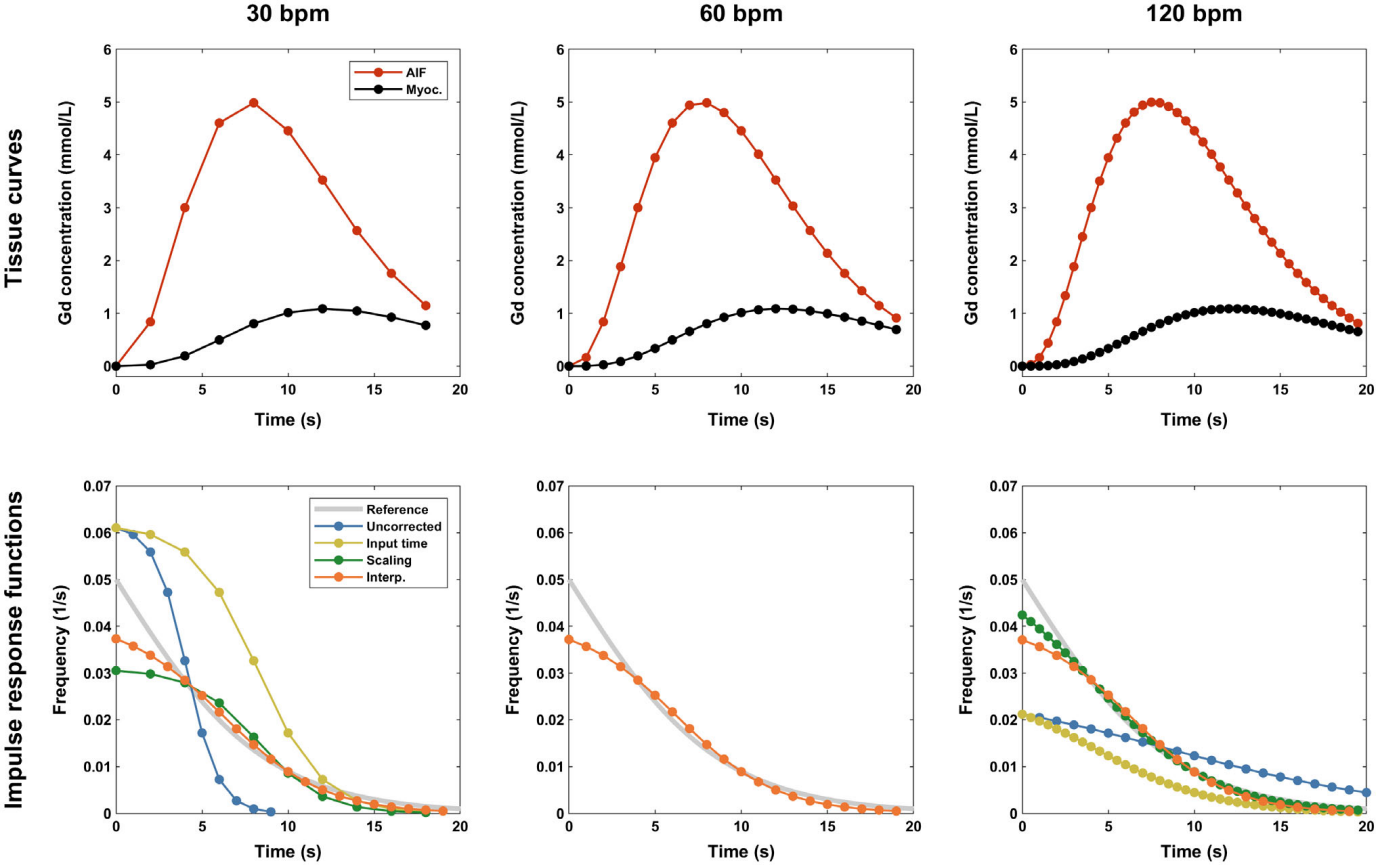
Impact of temporal resolution on myocardial perfusion quantification. The same noise‐free reference arterial and tissue curves were used to demonstrate the effect of three temporal resolutions for a fixed myocardial blood flow of 3 mL/g/min. Curves were analyzed using Fermi function‐constrained deconvolution based on a 20‐second temporal window to estimate the impulse response functions at the bottom row. If quantification is performed without accounting for temporal resolution (blue lines), the resulting functions differ in width and amplitude. Using the input time vector corrects the width of the functions but leads to the same erroneous blood flow (yellow lines), unless this is scaled by the inverse of the sampling interval to partially restore the correct value (green lines). Interpolation of curves to a fixed sampling rate (here used 60 bpm) ensures that blood flow is similar for all input sampling rates, with its accuracy depending on numerical errors associated with the interpolated rate (orange lines). All impulse response functions overlap for the input heart rate (HR) of 60 bpm.

The reference curves were resampled at five temporal resolutions to mimic data sampling at five different physiological HR (here called “input HR”): 30 bpm, 60 bpm, 90 bpm, 120 bpm, and 150 bpm (see Fig. 1, for example curves). Random temporal shifting within ±0.5 seconds was added to simulate errors in the detection of the tracer arrival time in the arterial and myocardial regions. Finally, random Rician noise was added to simulate typical noise levels reported for saturation recovery gradient echo sequences.^23^, ^24^ For each combination of MBF and HR, 1000 random realizations were produced for a total of 25,000 pairs of AIF and tissue curves. Simulations were performed using MATLAB (version 2019b; MathWorks®, Natick, Massachusetts, USA).

### Phantom Study

Dual‐bolus perfusion scans were acquired using a validated hardware phantom with a synthetic multicapillary myocardium.^25^, ^26^ The phantom was scanned on a 3‐T system (Biograph mMR; Siemens Healthcare, Erlangen, Germany) equipped with a 12‐channel anterior/posterior phased‐array coil system. One slice was acquired over the synthetic myocardium for a fixed reference MBF of 3 mL/g/min. Scanning was repeated three times for each of three different input HR simulated with retrospective ECG triggering (60 bpm, 90 bpm, and 120 bpm). A saturation recovery spoiled gradient echo sequence with fixed parameters was used: TR = 2.1 msec, TE = 1.1 msec, saturation recovery time = 100 msec, flip angle = 10°, pixel bandwidth = 1002 Hz, field of view = 280 × 210 mm^2^, acquired/reconstructed resolution = 1.5 × 1.5 mm^2^, slice thickness = 8 mm, and parallel acceleration factor = 2. Matching the clinical dual‐bolus MR perfusion protocol used in our institution, a dilute and a neat CA bolus with concentrations 0.0075 mmol/kg and 0.075 mmol/kg, respectively (Gadobutrol, Gadovist®, Bayer AG, Leverkusen, Germany) were injected in the vena cava of the phantom at 4 mL/sec using an injector pump (Spectris Solaris, Medrad®, Bayer AG). Each bolus was followed by a 25‐mL saline flush.

### Clinical Study

A retrospectively acquired dataset of 30 patients (male, *n* = 17; age median: 61 and range: 28 years–89 years) with (16, 53%) or without (14, 47%) CAD who were referred for a dual‐bolus MR perfusion study as part of their clinical care was used. Patients were scanned on a 3‐T system (Achieva TX; Philips Healthcare, Best, The Netherlands) equipped with a 32‐channel cardiac phased‐array coil. Three short‐axis slices through the basal, mid, and apical left ventricular cavity were acquired to assess myocardial perfusion during vasodilatory stress and at rest using an ECG‐triggered saturation recovery spoiled gradient echo sequence, with typical parameters: TR = 2.2 msec, TE = 1.0 msec, saturation recovery time = 100 msec, flip angle = 15°, pixel bandwidth = 1642 Hz, field of view = 380 × 360 mm^2^, acquired/reconstructed resolution = 2.6 × 2.6 mm^2^/1.3 × 1.3 mm^2^, slice thickness = 10 mm, and parallel acceleration factor = 1.8. The contrast injection protocol was identical to the phantom study. Two proton density images per slice were acquired at the beginning of each acquisition for estimating the coil sensitivity profile. Stress perfusion imaging was performed during adenosine‐induced hyperemia (140 μg/kg/min). Following a minimum delay of 10 minutes, a rest perfusion scan was acquired.

### Data Analysis

All data were analyzed to measure MBF with and without correction for temporal resolution. As described in the Supporting Information, quantification refers to the process of signal discretization, fitting and/or evaluation of the IRF for MBF estimation, which assume that the sampling interval of the signals (Δt) is fixed. Reliable quantification of MBF requires normalization by scaling by the inverse of Δt [equation (5), Supporting Information].

#### SIMULATIONS

For no correction for temporal resolution, the sampling rate and therefore Δt were completely ignored, which is equivalent to analysis with an assumed Δt = 1 second (input HR = 60 bpm). Additionally, no data interpolation was applied. For correction for temporal resolution, three different approaches were examined:

1. Analysis with the use of the input time vector for quantification (eg, *t* = 0, 2, 4, … seconds for input HR = 30 bpm) but omission of Δt for MBF normalization and no data interpolation (referred to as “input time correction”). This is the standard approach in tracer‐kinetic modeling.
2. Analysis with the use of the input time vector for quantification and Δt for MBF normalization, but no data interpolation (referred to as “MBF scaling correction”).
3. Analysis with data interpolation to a fixed temporal resolution and use of the interpolated time vector and Δt for quantification and MBF normalization, respectively (referred to as “interpolation correction”). For simplicity, an interpolated HR of 60 bpm (Δt = 1 second) was used here but higher temporal resolutions were subsequently examined, as described below. Four common interpolation algorithms were compared: linear, piecewise cubic Hermite,^27^ piecewise cubic spline, and smoothing cubic spline polynomials with a smoothing parameter value of 0.9.^28^

For interpolation correction, to determine the effect of different interpolated HR, the interpolation algorithm leading to the most accurate MBF estimation was used to interpolate data to even higher resolutions of up to Δt = 67 msec (interpolated HR = 900 bpm). MBF was normalized by the corresponding final Δt. The computational time required to process the simulated data across different temporal resolutions was also recorded. Processing was performed on a dedicated workstation (Intel Xeon W‐2175 CPU, 2.50 GHz, 256‐GB RAM).

To assess the impact of temporal resolution on quantification methods with differing mathematical derivation of MBF and algorithmic implementation, three methods were used: Fermi function‐constrained deconvolution,^29^, ^30^ truncated singular value decomposition (TSVD) with Tikhonov regularization,^31^, ^32^ and one‐compartment modeling.^33^ All methods are described in detail in the Supporting Information. Fermi function‐constrained deconvolution and TSVD estimate MBF from the IRF, whereas one‐compartment modeling estimates MBF directly as a free parameter during fitting. Comparing quantification methods directly with respect to their accuracy in MBF estimation was out of the scope of this study, as simulated data were generated based on one method alone.

Finally, to examine the influence of the length of the temporal window of the AIF and tissue curves, analyses were repeated for 20 seconds and 100 seconds; these correspond to a typical window including only the first pass of the CA^19^, ^34^ and a more ideal window capturing a long portion of the CA clearance, respectively.

#### PHANTOM STUDY

In phantom data, the AIF was extracted from a region of interest manually placed in the aorta of the perfusion phantom, as described previously.^26^ The AIF was used to quantify MBF at the pixel level using Fermi function‐constrained deconvolution alone (see Supporting Information), and the mean value was obtained from the maps over the whole myocardial area.

MBF was measured with and without correction for temporal resolution with the same three approaches used in Monte Carlo simulations, as described above. For MBF scaling correction, the input Δt for each scan (which was fixed across images) was derived automatically based on the acquisition time recorded in the header of perfusion DICOM images. For interpolation correction, interpolation was performed using the optimal algorithm and at the optimal temporal resolution determined in the simulation study.

#### CLINICAL STUDY

Perfusion images first underwent motion correction and were corrected for coil sensitivity using the acquired proton density maps.^35^ Then, pixelwise MBF was quantified using an automated pipeline that detects the left ventricle in the basal slice, exports the AIF, and prepares the curves for quantification.^35^ MBF was estimated using Fermi function‐constrained deconvolution alone (see Supporting Information). The mean (global) MBF in all three short‐axis slices was measured at both rest and stress, which also allowed estimation of global MPR as the stress‐to‐rest ratio of MBF.

MBF was measured with and without correction for temporal resolution similar to the phantom study. For MBF scaling correction, the median Δt across the whole scan was used due to the variable patient HR and to ignore any potential outlying values due to ECG mistriggering. For interpolation correction, interpolation was performed after coil sensitivity correction and before quantification using the optimal algorithm and at the optimal temporal resolution determined in the simulation study.

### Statistical Analysis

The normality of MBF and MPR was assessed using the Shapiro–Wilk test. Normally distributed measurements were summarized using the mean and standard deviation (SD), while non‐normally distributed measurements were summarized using the median and range. In the simulation study, the accuracy in MBF was assessed using the percentage error (PE). For plotting absolute MBF and its PE, piecewise cubic spline fitting was used to generate a continuous curve for each reference MBF across the input HR range. In the clinical study, rest MBF, stress MBF, and MPR were compared between approaches using one‐way repeated measures analysis of variance with Bonferroni correction for post hoc pairwise comparisons. The test was two‐tailed and statistical significance was set at *P* < 0.05. The agreement between the most accurate approach (as determined in the phantom study) and other approaches for correcting for temporal resolution was examined using Bland–Altman analysis. Statistical analysis was performed in SPSS® (version 25.0; IBM Corp., Armonk, New York, USA).

## Results

### Simulations

Figure 1 demonstrates the impact of temporal resolution using example simulated data without random temporal shifting and noise, analyzed in these examples using Fermi function‐constrained deconvolution. Without correction, higher temporal resolutions increase the width and decrease the amplitude of the estimated IRF, causing a corresponding decrease in MBF. The width of IRF is restored following input time correction, but its amplitude and therefore MBF remain incorrect. The amplitude of IRF can be partially restored by either MBF scaling correction or interpolation correction. While the accuracy of MBF scaling correction depends on the temporal resolution of the input data, interpolation correction provides the same estimates independent of the input resolution (in this example, the IRF maximum following interpolation is 0.037 seconds^−1^ at all three temporal resolutions).

#### NO CORRECTION FOR TEMPORAL RESOLUTION

**FIGURE 2:**
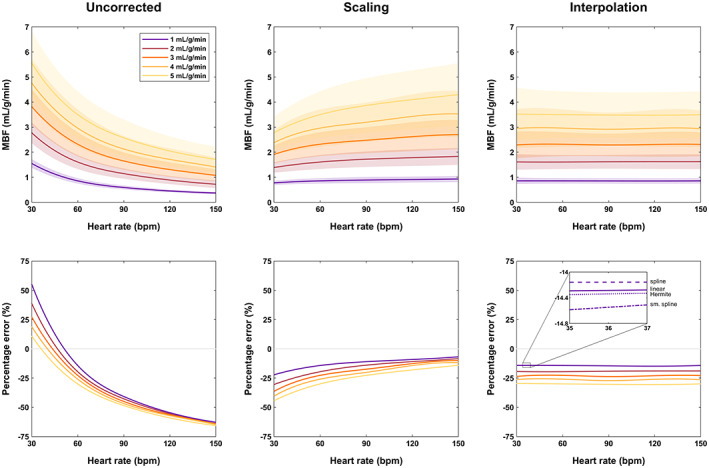
Estimated myocardial blood flow in simulated data for Fermi function‐constrained deconvolution. Plots show blood flow (top row) and its percentage error (bottom row) for three approaches: no correction for temporal resolution (left column), myocardial blood flow (MBF) scaling correction (center column), and data interpolation to 60 bpm (right column); 20 seconds were used for quantification. The lines indicate the mean estimate and the shaded areas the SD (except for percentage error for clarity), with cubic spline fitting used to generate a continuous curve for each perfusion rate over all heart rates. The inset plot for interpolation shows the differences between interpolation algorithms for 1 mL/g/min reference blood flow.

Without correction for temporal resolution, MBF was not accurately estimated by each of the three quantification methods (Fig. 2 and Figures S1 and S2). There was a trend toward overestimation of MBF at lower input HR and underestimation at higher input HR.

Specifically, for Fermi function‐constrained deconvolution, PE ranged between 55.4% and −62.7% for reference 1 mL/g/min across the tested range of input HR (30 bpm–150 bpm respectively; Fig. 2). For reference 5 mL/g/min, PE ranged between 11.25% and −65.7%. For TSVD, PE ranged between 79.2% and −63.2% for reference 1 mL/g/min, and between 6.7% and −73.0% for reference 5 mL/g/min (Figure S1). For one‐compartment modeling, PE ranged between 60.9% and −60.8% for reference 1 mL/g/min, and between 36.3% and −59.0% for reference 5 mL/g/min (Figure S2).

#### CORRECTION FOR TEMPORAL RESOLUTION

Input time correction led to the same MBF values as in uncorrected quantification for all three quantification methods (no difference in values in the left column in Fig. 2 and Figures S1 and S2), except for very small reference MBF and HR in one‐compartment modeling, for which a small difference in estimated MBF was observed (1 mL/g/min: PE range 71.6% to −60.8%; PE for 5 mL/g/min was identical to uncorrected quantification; Figure S2).

MBF scaling correction reduced errors for all quantification methods, but their pattern was different; MBF was more underestimated for lower HR and higher reference MBF. For Fermi function‐constrained deconvolution, PE ranged between −22.2% and −6.8% for reference 1 mL/g/min and between −44.4% and −14.0% for reference 5 mL/g/min (Fig. 2). For TSVD, PE ranged between −10.4% and −8.0% for reference 1 mL/g/min, and between −46.7% and −32.5% for reference 5 mL/g/min (Figure S1). For one‐compartment modeling, PE ranged between −17.7% and −1.9% for reference 1 mL/g/min, and between −31.9% and 2.4% for reference 5 mL/g/min (Figure S2).

Interpolation correction decreased MBF variability across the full range of input HR for all interpolation algorithms and for all quantification methods. For example, for cubic spline interpolation: Fermi function‐constrained deconvolution PE ranged between −14.2% and −14.2% for reference 1 mL/g/min, and between −29.6% and −30.0% for reference 5 mL/g/min (Fig. 2); TSVD PE ranged between −7.3% and −10.7% for reference 1 mL/g/min, and between −38.2% and −38.6% for reference 5 mL/g/min (Figure S1); one‐compartment modeling PE ranged between −9.6% and −9.2% for reference 1 mL/g/min, and between −13.0% and −13.4% for reference 5 mL/g/min (Figure S2). MBF errors for an input HR of 60 bpm were matched for uncorrected quantification and all correction approaches, given that the interpolated HR for interpolation correction was also 60 bpm; this is also demonstrated in Fig. 1.

**FIGURE 3:**
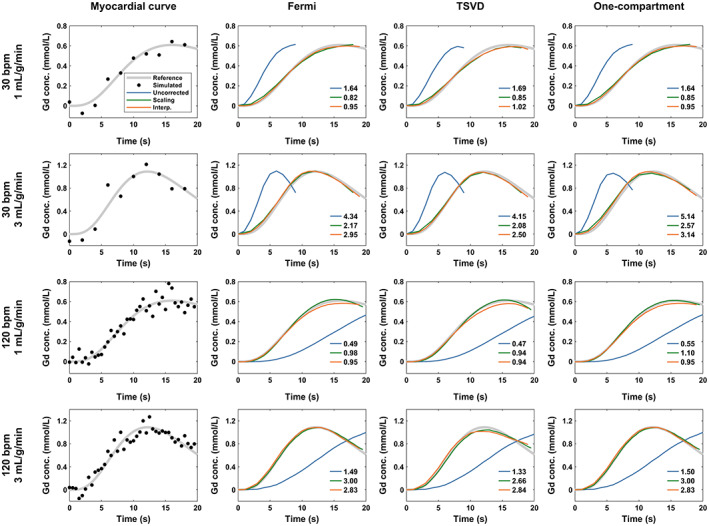
Example simulated myocardial curves and estimated myocardial blood flow for all quantification methods. The plots show a simulated curve (black points) for two different heart rate (HR) and two reference myocardial blood flow (MBF) values, as well as the fitted curves using each quantification method. The reference noise‐free curves are shown in gray. The measured blood flow (in mL/g/min) is given in each plot. Note that the fitted curves for correction by flow scaling (green lines) have the correct amplitude only if the arterial input function is scaled by Δt, which is equivalent to scaling flow by the inverse of Δt.

Figure 3 compares the fitting results for all quantification methods for example curves simulated with two reference MBF values and two input HRs. No correction for temporal resolution leads to fitting of myocardial curves poorly matching the reference curves, the width of which depends on the temporal resolution of the input data. MBF scaling and interpolation corrections improve MBF accuracy for all quantification methods. Since input time correction leads to estimation of the same MBF as uncorrected quantification, it was excluded from further simulation analyses as well as the phantom and clinical studies.

#### COMPARISON OF INTERPOLATION ALGORITHMS

**FIGURE 4:**
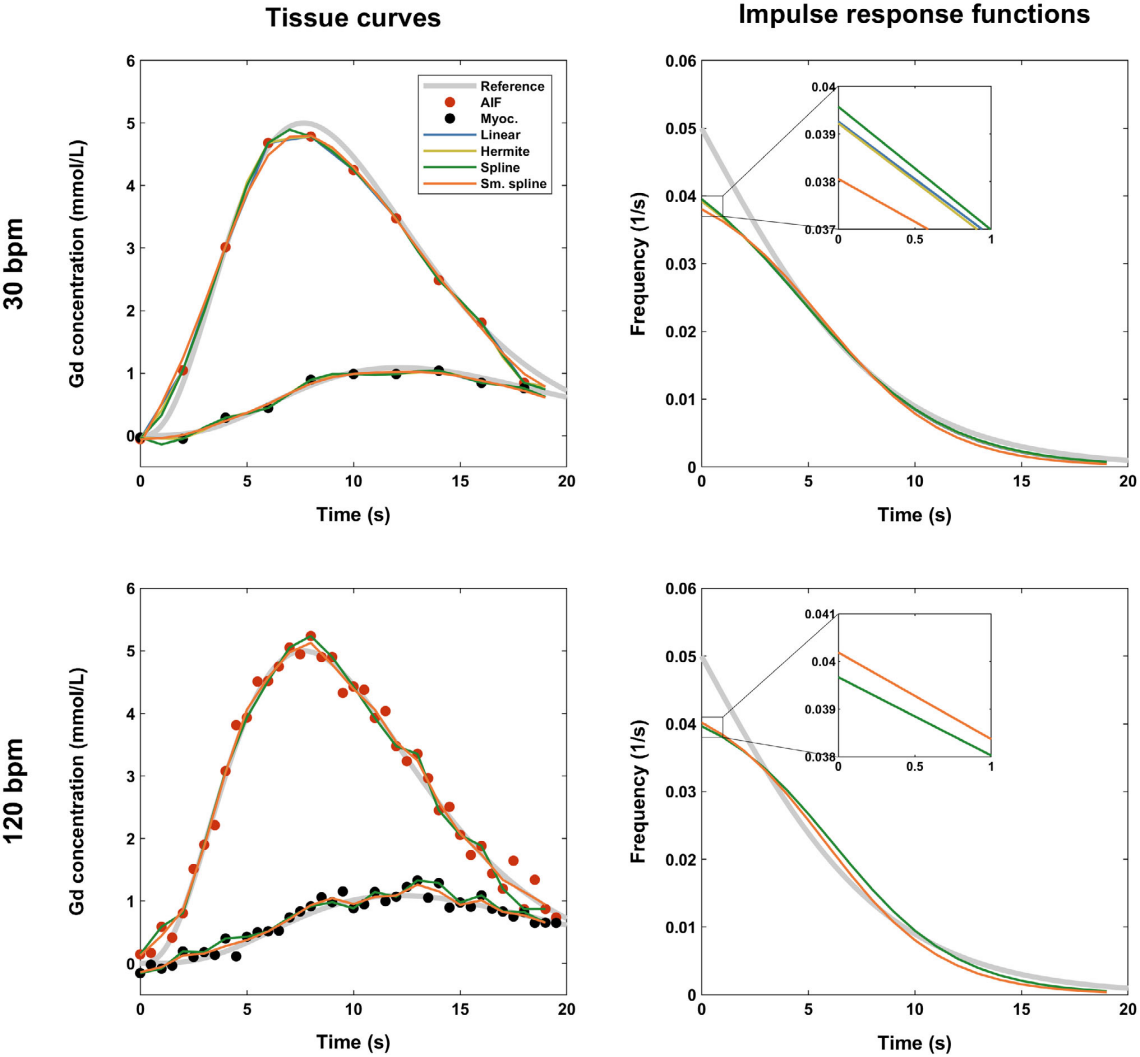
Comparison of signal interpolation methods for myocardial perfusion quantification. Plots show two example pairs of arterial and tissue curves (left column) for two different input heart rates (HRs) and a fixed blood flow of 3 mL/g/min, as well as their corresponding impulse response functions (right column). Fermi function‐constrained deconvolution following curve interpolation to a fixed rate of 60 bpm was used. The interpolation method has a small impact on perfusion quantification. Since the input HR of 120 bpm is a multiple of the interpolated HR, all interpolation methods except the smoothing spline led to identical and overlapping response curves. The smoothing spline partially filters the data, which may lead to errors as it can suppress important features such as contrast enhancement upslopes and peaks.

The four tested interpolation algorithms led to nearly identical MBF measurements at all simulated scenarios. For example, for Fermi function‐constrained deconvolution and reference 1 mL/g/min, PE ranged between −14.3% and −14.1% for linear interpolation, between −14.4% and −14.1% for cubic Hermite interpolation, between −14.2% and −14.2% for cubic spline interpolation, and between −14.8% and −14.2% for smoothing cubic spline interpolation. Interpolation with cubic spline polynomials had marginally the highest accuracy for HR < 60 bpm (inset in Fig. 2 and Figures S1 and S2) but was not better for HR ≥ 60 bpm. Interpolation with smoothing cubic splines led to the most variable quantification. Figure 4 shows the performance of all algorithms on example simulated curves.

#### IMPACT OF INTERPOLATED HR

**FIGURE 5:**
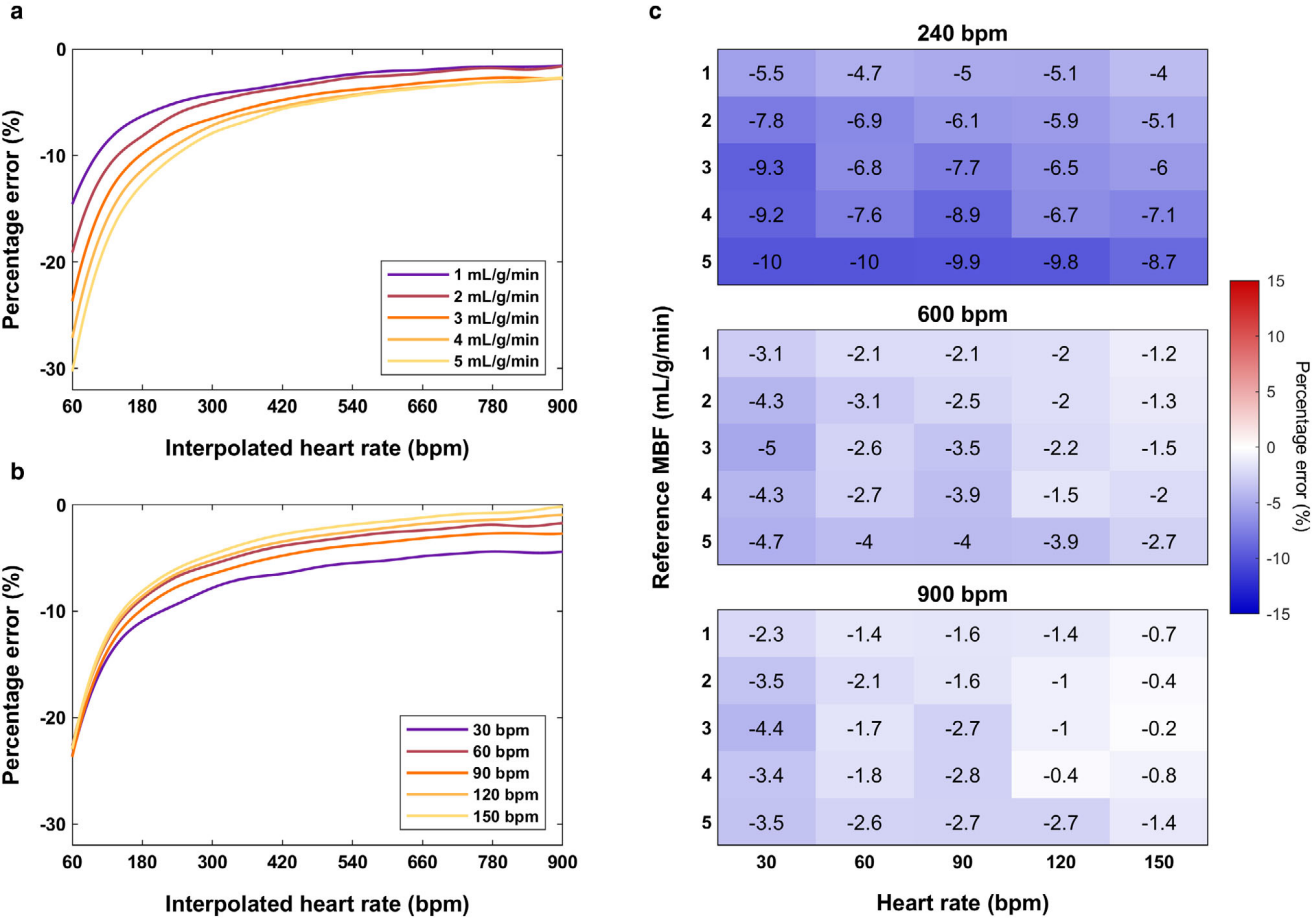
Error in the estimated myocardial blood flow in simulated data following cubic spline interpolation to various temporal resolutions. Fermi function‐constrained deconvolution was used. Percentage error in blood flow for (**a**) the input heart rate of 90 bpm and all reference flow rates (1–5 mL/g/min) and (**b**) reference value 3 mL/g/min and all input heart rates (30–150 bpm). The SD is not shown for clarity. Cubic spline fitting was used to generate a continuous curve for each flow rate in (a) and input heart rate in (b). The error for all 25 combinations of the two parameters is shown in (**c**) for 240 bpm (all errors ≤10%), 600 bpm (all errors ≤5%), and 900 bpm (maximum tested) interpolated heart rate above which most errors remained fixed. Corresponding MBF precision and processing time measurements are shown in Figure S3.

Cubic spline polynomials were used to assess the impact of interpolation to temporal resolutions higher than Δ*t* = 1 second with Fermi function‐constrained deconvolution (Fig. 5). The accuracy in MBF estimation increases with the interpolated temporal resolution, with MBF errors approaching zero faster for lower reference MBF and higher input HR. Errors for large reference MBF and low input HR practically never reach zero, even at an interpolated HR of 900 bpm. The minimum interpolated HR for which the maximum absolute error for all simulated data is 10% and 5% is 240 bpm and 600 bpm, respectively (Fig. 5c).

In contrast, the precision in MBF estimation decreases with the interpolated temporal resolution (Figure S3). For a typical hyperemic input HR of 90 bpm and a reference flow rate of 3 mL/g/min, the SD of MBF was 0.51 mL/g/min when measured at the native HR and increased to 0.62 mL/g/min at 240 bpm (22% increase) and to 0.66 mL/g/min at 600 bpm (30% increase). The mean processing time increased from 7.6 seconds at 90 bpm to 8.7 seconds at 240 bpm (14% increase) and to 20.2 seconds at 600 bpm (165% increase; Figure S3). The processing time reached 45.9 seconds at the maximum tested interpolated HR of 900 bpm (502% increase).

#### IMPACT OF TEMPORAL WINDOW FOR QUANTIFICATION

The pattern of MBF errors did not change for quantification with a longer temporal window of 100 seconds (Figure S4). For all correction approaches, the errors decreased for Fermi function‐constrained deconvolution and one‐compartment modeling. For interpolation correction, the largest absolute PE difference compared to the temporal window of 20 seconds was 6.6% and 14.7% for the two quantification methods, respectively. In contrast, errors increased for TSVD, with the largest absolute PE difference for interpolation correction being 6.0%.

### Phantom Study

The impact of temporal resolution on phantom MBF (reference 3 mL/g/min) was examined using Fermi function‐constrained deconvolution (Figure S5). Interpolation was performed at 240 bpm using cubic spline polynomials, based on the results of simulations. For uncorrected quantification, MBF decreased with increasing HR [2.81 (2.76–3.06) mL/g/min for 60 bpm, 1.85 (1.31–2.22) mL/g/min for 120 bpm]. MBF scaling correction led to less accurate and more variable estimates [3.72 (2.61–4.43) mL/g/min for 120 bpm] compared to interpolation correction [2.81 (2.03–2.91) mL/g/min for 120 bpm].

### Clinical Study

**FIGURE 6:**
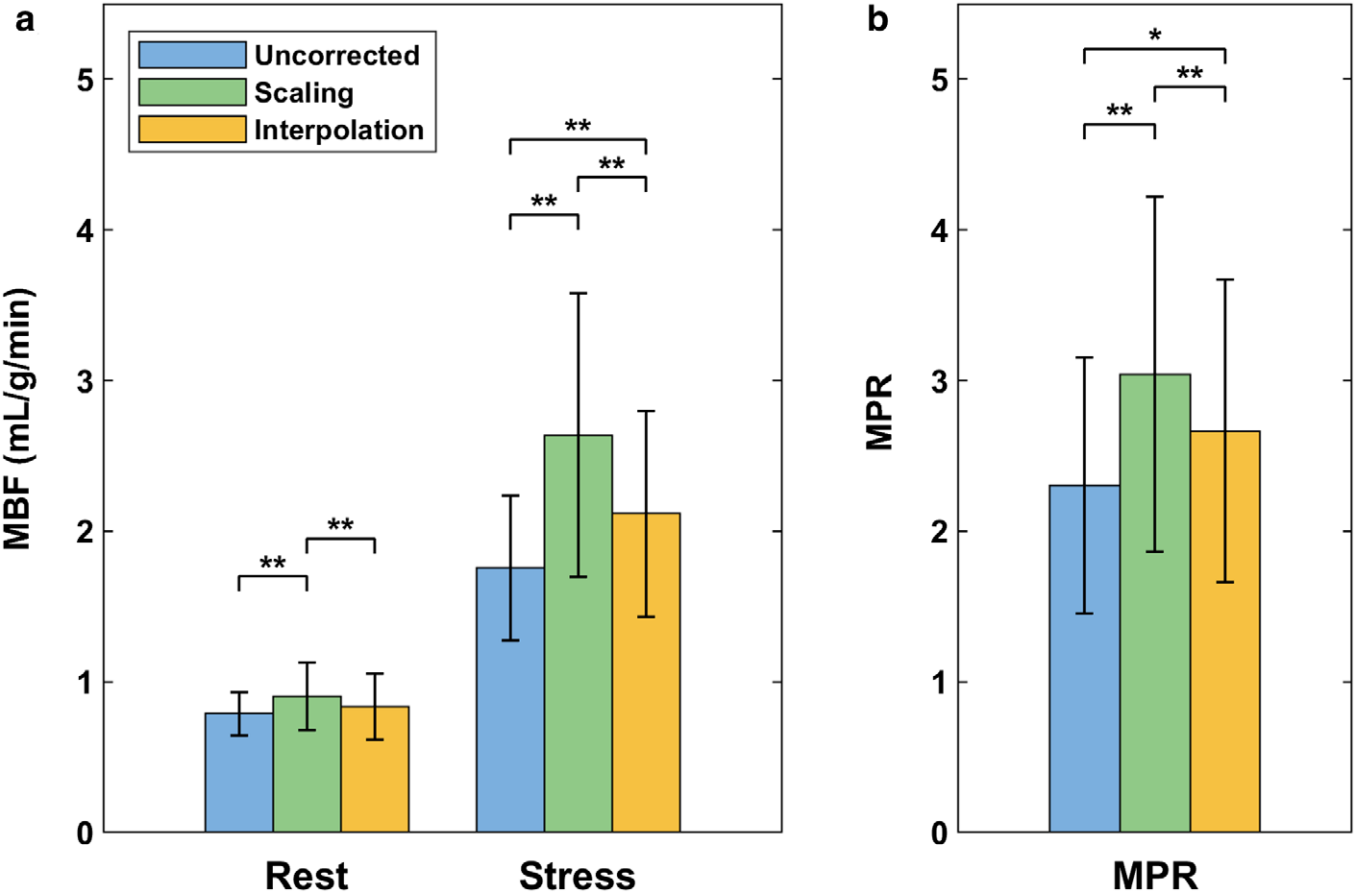
Global myocardial blood flow (**a**) and myocardial perfusion reserve (**b**) in patients quantified with and without correction for temporal resolution. *P*‐values are based on one‐way repeated measures analysis of variance with Bonferroni correction for post hoc pairwise comparisons; single asterisk (*) and double asterisks (**) indicate *P* < 0.05 and *P* < 0.001, respectively.

The mean patient HR was 69 ± 13 bpm at rest and 91 ± 17 bpm during stress. Identical to the phantom study, analysis of clinical data was performed using Fermi function‐constrained deconvolution and interpolation was performed at 240 bpm using cubic spline polynomials. Rest MBF (uncorrected: 0.79 ± 0.14 mL/g/min, MBF scaling: 0.90 ± 0.22 mL/g/min, interpolation: 0.83 ± 0.22 mL/g/min), stress MBF (uncorrected: 1.76 ± 0.48 mL/g/min, MBF scaling: 2.64 ± 0.94 mL/g/min, interpolation: 2.12 ± 0.68 mL/g/min), and MPR (uncorrected: 2.31 ± 0.85, MBF scaling: 3.04 ± 1.18, interpolation: 2.66 ± 1.00) were all significantly different between uncorrected and corrected quantification (Table 1 and Fig. 6). Rest MBF between only uncorrected quantification and interpolation correction was not significantly different (*P* = 0.231). Stress MBF and MPR, however, were significantly different between all pairs. Based on the SD of stress MBF and MPR, interpolation correction led to less variable estimates compared to MBF scaling correction. Bland–Altman analysis showed that uncorrected quantification underestimates MBF and MPR compared to interpolation correction [bias: −0.20 mL/g/min, 95% confidence interval (CI): −0.93 mL/g/min to 0.53 mL/g/min; bias: −0.36, 95% CI: −1.68 to 0.96, respectively; Figure S6), while MBF scaling correction overestimates both metrics (bias: 0.30 mL/g/min, 95% CI: −0.53 mL/g/min to 1.12 mL/g/min; bias: 0.38, 95% CI: −0.60 to 1.35, respectively).

**TABLE 1:** Clinical Myocardial Blood Flow and Perfusion Reserve with and Without Correction for Temporal Resolution

Method	Uncorrected	Scaling	Interpolation	*P*‐value
Rest MBF (mL/g/min)	0.79 ± 0.14	0.90 ± 0.22	0.83 ± 0.22	<0.001
Stress MBF (mL/g/min)	1.76 ± 0.48	2.64 ± 0.94	2.12 ± 0.68	<0.001
MPR	2.31 ± 0.85	3.04 ± 1.18	2.66 ± 1.00	<0.001

Data expressed as mean ± SD. Pairwise comparisons are shown in Fig. 6.

MBF = myocardial blood flow; MPR = myocardial perfusion reserve.

**FIGURE 7:**
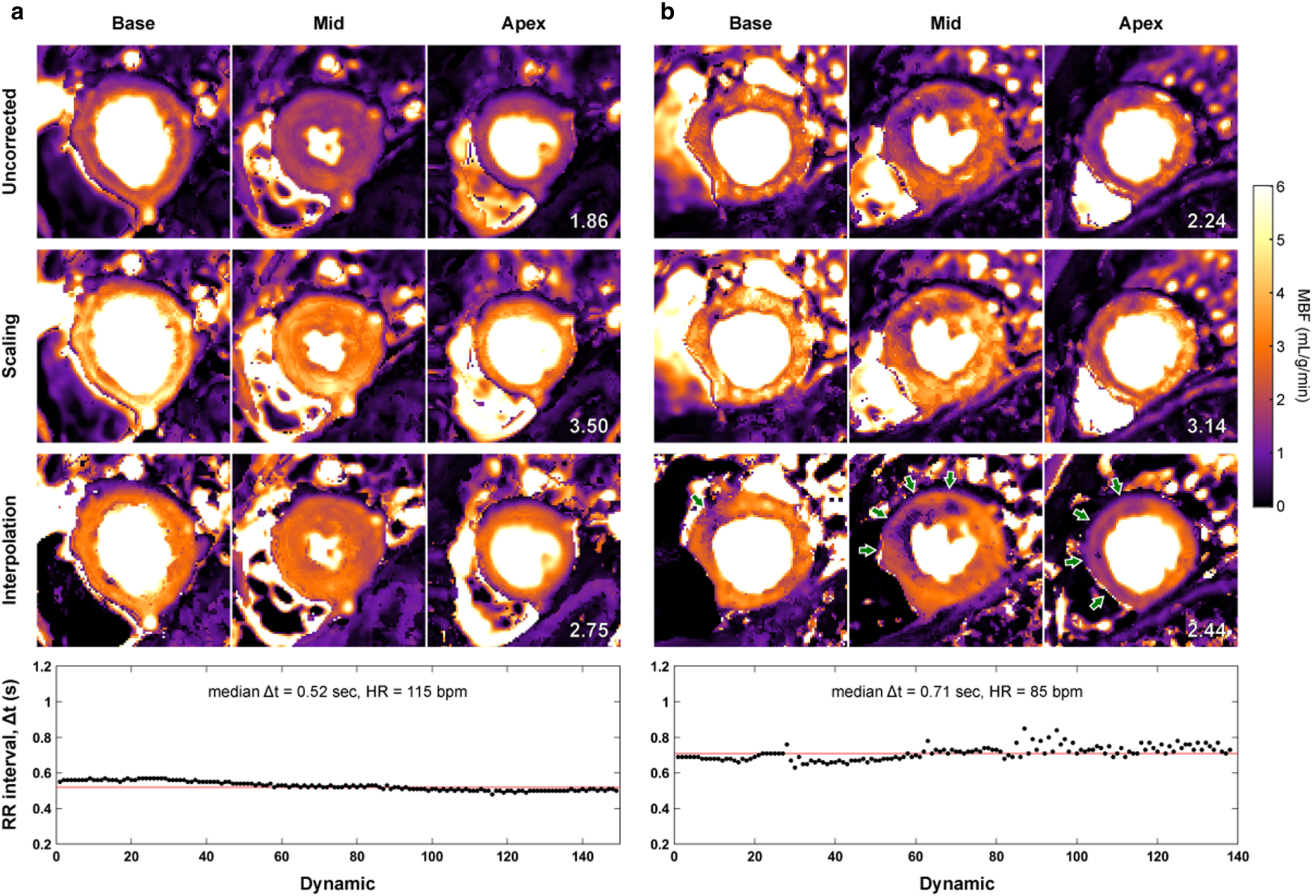
Effect of temporal resolution on pixelwise perfusion maps. Stress myocardial blood flow maps were generated for a patient without coronary artery disease (**a**) and a patient with single‐vessel disease (**b**). The plot of the corresponding sampling interval per dynamic, Δt, is provided at the bottom. The mean blood flow (in mL/g/min) in all three slices is given in the apical slice. Quantification without correction for temporal resolution estimated a low stress blood flow, particularly in (a) as the patient's heart rate is faster. Myocardial blood flow (MBF) scaling correction increased flow in both ischemic and remote myocardium. Data interpolation to 240 bpm also led to an increased flow, but without affecting the delineation of perfusion defects [green arrows in (b)].

Figure 7 demonstrates the effect of the various correction approaches on pixelwise stress MBF. MBF scaling correction leads to MBF estimates within the expected physiological range but increases flow in both ischemic and remote myocardium. Alternatively, interpolation correction increases mean MBF without affecting the delineation of perfusion defects.

## Discussion

This study has shown the impact of sampling rate and temporal resolution on myocardial perfusion quantification. Findings in the simulation study were corroborated by phantom data that mimic more closely clinical perfusion imaging while allowing reproducible control of the reference MBF and input HR. Furthermore, in concordance with simulations, rest MBF, stress MBF, and MPR in patients were all significantly different between quantification with and without correction for temporal resolution. Quantification following data interpolation was found to be superior to MBF scaling as it decreases the variability across different temporal resolutions.

Temporal resolution is a parameter inherently linked to the quantification of MBF. The arterial and myocardial signals are discretized before being used in deconvolution analysis for the measurement of MBF. While it is known that the sparsity of signals used in such problems impacts the frequency distribution of the solution,^9^ its impact on myocardial perfusion quantification is unclear. The mechanism relies on the fact that densely sampled signals will consist of more frequency components than sparsely sampled ones.^7^ As a result, the estimated IRF representing the frequency distribution of CA transit times will be equivalently dense. The sum of the IRF components is governed by the ratio of the two deconvolved signals which largely remains constant at different sampling rates. However, a change in the sampling frequency and therefore the number of IRF frequency components will cause a change in the IRF amplitude to preserve the sum of the components. If data are not normalized for temporal resolution, analysis for two different sampling rates will lead to two differently scaled IRF curves and consequently MBF values.

Despite the theoretical expectation that scaling of MBF by the inverse of Δt (which, in deconvolution analysis, is equivalent to scaling AIF by Δt) can restore its correct value, our simulations demonstrate that such a “corrected” MBF has a nonlinear relationship with the sampling rate and severe underestimations occur at low rates. We can attribute this observation to the fact that low sampling rates can affect not just the amplitude but also the overall shape of deconvolution‐based IRF curves. Specifically, inverse sigmoidal IRF shapes with initial plateaus are favored toward low rates, causing a decrease in the estimated MBF. In contrast, exponential decay‐like shapes are favored toward higher rates. A similar effect was observed for both Fermi function‐constrained deconvolution and TSVD, whereas no dependence on model formulation, initial parameter values, or the settings of the optimization algorithms was detected. Our hypothesis is that low sampling rates lead to discretization errors in MBF quantification because data insufficiency makes the problem poorly scaled and introduces erroneous solutions. We observed that discretization errors prevail over well‐known quantification errors arising from potential undersampling of the AIF peak at low sampling rates that could overestimate perfusion,^36^ with MBF normalization alone not being able to account for them. Noise further contributes to the observed MBF underestimation, with expectedly a bigger impact on larger reference MBF and lower input HR. Finally, our results indicate that absolute PE is larger for large reference MBF values, because sharper myocardial peak enhancements may be more severely affected by noise or be more poorly sampled. At low MBF values, tissue curves are more dispersed and therefore more adequately sampled over the same temporal window.

On the other hand, data interpolation to a fixed temporal resolution prior to quantification accompanied by MBF normalization leads to a nearly constant PE across all input HR. This is because interpolation ensures that the signals contain the same frequency components independent of the rate by which they were originally sampled, stabilizing discretization errors and thus MBF quantification. However, depending on the rate to which the data are interpolated to, measurements will still be impacted by a systematic error associated with that rate; for example, interpolation to 60 bpm carries the same errors measured at that input rate when no interpolation is performed. We can therefore infer that interpolation to higher temporal resolutions increases the accuracy in MBF quantification, as the numerically approximated solution for the sampled discrete signals approaches the exact solution for the real continuous signals.^7^ This was indeed the case, with interpolation to 240 bpm decreasing overall absolute errors to 10% or less and interpolation to 600 bpm to 5% or less. No interpolation rate can provide perfect accuracy as this is limited by the input HR and SNR, which combined can lead to permanent degradation of the signals.

An important practical consideration is that an increase in the interpolated sampling rate may result in unwanted oscillations in the signals that can impact processing steps such as baseline correction and identification of timing points, affecting the precision of pixelwise MBF. Furthermore, we observed an increase in processing time with the interpolated sampling rate, which can limit its application in the clinical setting where the generation of perfusion maps in a timely manner is important. Therefore, a good sampling rate to interpolate data to must be a compromise between accuracy, precision, and speed. It was found that an interpolated HR of 240 bpm decreases PE ≤10% and has a small impact on MBF precision and processing speed. Furthermore, any interpolation algorithm other than smoothing cubic splines practically has the same performance. The recommendation applies to Fermi function‐constrained deconvolution and one‐compartment modeling, whereas the larger absolute errors for TSVD imply that the 10% absolute error margin would potentially require higher interpolated rates. Model‐independent deconvolution produces IRF curves that are not necessarily monotonically decreasing and often impose substantial smoothing on their peak, hence underestimating perfusion.^14^ Therefore, such methods should be subjected to further optimization to carefully select suitable truncation and/or regularization parameters for the specific interpolation strategy used.

The length of the temporal window does not affect the pattern of errors measured for corrected or uncorrected quantification but affects the overall MBF accuracy, in agreement with previous studies.^34^ This suggests that longer windows would potentially alter the minimum interpolated HR for which quantification error approaches zero. However, in deconvolution‐based methods, the arterial and myocardial signals must be cropped to remove baseline dynamics and CA recirculation components which, if included in quantification, can increase MBF errors. Further work is needed to determine the optimal interpolated HR for other quantification methods that make use of different parts of the signals.^37^, ^38^ As only the Fermi function was used to generate all simulated tissue curves, our aim was not to draw conclusions with regard to the superiority of one quantification model over the other, hence we limited our assessment to the impact of temporal resolution on each individual method.

In this study, correction for temporal resolution by MBF scaling and data interpolation was also applied to the quantification of phantom and clinical perfusion datasets. Clinical rest MBF was found to be significantly different between MBF scaling and uncorrected quantification, but there was no significant difference between data interpolation and uncorrected quantification. This could be due to the acquisition of rest data with lower sampling rates closer to the reference 60 bpm assumed by uncorrected quantification. In contrast, stress MBF was significantly different between all three approaches as stress images are acquired with higher sampling rates that carry the risk of larger MBF errors when no correction is applied. As a result of the significant variation in stress MBF, we also detected significant differences between MPR for corrected and uncorrected quantification.

### Limitations

In the clinical study, we used the median Δt across the whole dynamic series for MBF scaling. This may not be the optimal strategy, considering that patient's HR and consequently sampling rate can be different for different parts of the signal that contribute in variable extents to the measured MBF. The AIF peak and myocardial upslope only account for very few and differing dynamics, therefore determining a representative sampling interval may not only be mathematically unstable but also algorithmically challenging.^7^ This is an additional argument for data interpolation to a fixed temporal resolution, corroborated by the smaller MBF variability measured in this study. We used two different 3 T scanners for phantom and clinical imaging; however, discretization errors arising from sparse sampling rates are linked to the quantification process and are not known to be dependent on the scanner vendor or the perfusion sequence. Finally, there was no reference diagnosis for included patients hindering an assessment of the impact on diagnostic accuracy, but could be the focus of future work.

### Conclusion

Incorporation of a correction for temporal resolution of perfusion data is essential to ensure accurate measurement of absolute MBF and MPR. This can be performed by either scaling MBF by the inverse of the sampling interval or interpolating the dynamic perfusion data prior to quantification to a high temporal resolution. The latter is recommended as it reduces discretization errors and the variability in perfusion measurements across different temporal resolutions. Consequently, interpolation can assist in the pooling of data across patient populations with variable HR or studies employing different data sampling strategies. Future developments in image acquisition and reconstruction techniques with increased temporal resolution could reduce the errors associated with the loss of information from data undersampling in patients with low HR.

## Supporting information


**Appendix S1:** Supplementary Information
