# Supplementary material for: Impact of Temporal Resolution and Methods for Correction on Cardiac Magnetic Resonance Perfusion Quantification
Source: J Magn Reson Imaging. 2022 Mar 26;56(6):1707–19. doi: 10.1002/jmri.28180 (PMC9790572; doi:10.1002/jmri.28180)
Supplement: Supplementary file 1 — Appendix S1: Supplementary Information [file JMRI-56-1707-s001.docx]

**Supporting Information**

**Fundamentals of perfusion quantification**

Under the assumption that the coronary and myocardial circulation is a linear time-invariant system, injection of a non-diffusible contrast agent (CA) and measurement of its concentration over time allows derivation of myocardial blood flow (MBF) [1]. The process requires sampling of an arterial concentration typically referred to as the arterial input function, $C_{aif}\left( t \right)$, upstream to the myocardium, and the tissue concentration, $C_{myo}\left( t \right)$, at a location over the myocardium. These signals are related by convolution, a mathematical operation determining how $C_{aif}\left( t \right)$ is dispersed across the two sampling locations to become $C_{myo}\left( t \right)$.

$$C_{myo}\left( t \right)=C_{aif}\left( t \right)\otimes R_{F}\left( t \right)$$

$R_{F}\left( t \right)$ is a function specific to the system and describes the contribution of any infinitesimally small portion of the inlet to the total outflux from the myocardium. It is defined as the tissue’s impulse response function, the response (output) of the system when $C_{aif}\left( t \right)$ is an impulse—an ideal instantaneous bolus (i.e. a Dirac’s delta). For continuous time systems, the convolution equation can be stated as an integral:

$$C_{myo}\left( t \right)=\int_{0}^{t} \left. C_{aif}\left( \tau\right) \right.R_{F}(t-\tau)d\tau(1)$$

$R_{F}\left( t \right)$ also represents the probability distribution of the transit times of the CA molecules, meaning that the integral over any time interval is the probability that the transit time lies within that interval. Using the principles of the indicator-dilution theory [1,2], measurement of $R_{F}\left( t \right)$ allows quantification of MBF if one treats it as the tissue residue function, $R\left( t \right)$, scaled by flow $F$:

$$R_{F}\left( t \right)=F\cdot R(t)$$

$R\left( t \right)$ is a monotonically decreasing function giving the fraction of CA in tissue at time $t$. At $t=0$, all CA molecules remain in the tissue ($R\left( 0 \right)=1$) and therefore $R_{F}\left( 0 \right)=F$. As $F$ is obtained from a probability distribution, it represents an index of flow through the myocardium and must be corrected for the time scale and the density of the tissue to provide MBF with units of perfusion rate [3]. Practically, $F$ is often taken to be the maximum of $R_{F}(t)$ instead of $R_{F}(0)$ as the latter is sensitive to numerical and bolus dispersion errors [4,5].

Knowledge of $R_{F}\left( t \right)$ also permits measurement of myocardial blood volume (MBV) and mean transit time (MTT) [1]. The fractional volume where CA is diffusible is given by the area under $R_{F}\left( t \right)$:

$$V=\int_{0}^{\infty} R_{F}\left( t \right)dt$$

For an extracellular CA, $V$ corresponds to the vascular and extracellular spaces [6]. Normalizing $V$ by the myocardial density provides MBV as a ratio of CA dispersion volume to tissue mass. MTT, the mean of residence times of all CA molecules in the tissue, is the area under $R(t)$:

$$MTT=\int_{0}^{\infty} R\left( t \right)dt (2)$$

It follows that these quantities are related by the central volume principle:

$$F=\frac{V}{MTT}$$

In clinical dynamic perfusion imaging, the measured $C_{aif}\left( t \right)$ and $C_{myo}\left( t \right)$ are discrete and not continuous, sampled with a fixed or variable rate. Therefore, to estimate $R_{F}(t)$ the convolution integral in equation (1) is reformulated to a matrix equation:

$$C_{myo}\left( t \right)\approx\Delta t\sum_{i=0}^{j} C_{aif}\left( t_{i} \right)R_{F}(t_{j}-t_{i})$$

where $\Delta t$ is the sampling interval and which must be fixed to reduce numerical errors in the solution. The equation can be written in matrix form as

$$\left( \begin{matrix} C_{myo}(t_{1}) \\ C_{myo}(t_{2}) \\ \ldots\\ C_{myo}(t_{n}) \end{matrix} \right)\approx\Delta t\left( \begin{matrix} C_{aif}(t_{1}) & 0 & \ldots& 0 \\ C_{aif}(t_{2}) & C_{aif}(t_{1}) & \ldots& 0 \\ \ldots& \ldots& \ldots& \ldots\\ C_{aif}(t_{n}) & C_{aif}(t_{n-1}) & \ldots& C_{aif}(t_{1}) \end{matrix} \right)\left( \begin{matrix} R_{F}(t_{1}) \\ R_{F}(t_{2}) \\ \ldots\\ R_{F}(t_{n}) \end{matrix} \right)$$

This process is called discretization and converts an integral equation into a system of linear equations that can be solved numerically and provide an approximate discrete solution. The discretized form of $C_{aif}\left( t \right)$ is a lower-triangular Toeplitz matrix. The convolution equation can be solved iteratively for $R_{F}(t_{i})$ by the inverse process of deconvolution of $C_{aif}\left( t \right)$ and $C_{myo}\left( t \right)$. However, this process is very sensitive to noise and therefore the equation is typically solved as a least-squares problem instead. If we rewrite the last equation in short-hand vector notation,

$$\mathbf{M}=\mathbf{AR} (3)$$

the solution can be obtained by least-squares minimization of the residual norm

$$\hat{\mathbf{R}}=arg min\left. \left\| \mathbf{AR}\boldsymbol{-}\mathbf{M} \right\|^{2} \right. (4)$$

The elements of $\mathbf{A}$ can incorporate $\Delta t$, though the same MBF can be obtained by otherwise scaling the estimated $R_{F}\left( t \right)$ or MBF itself by the inverse of $\Delta t$ [7,8]. Overall, MBF can be estimated in typical units of perfusion rate (milliliters of blood per gram of tissue per minute) using

$$MBF=F\frac{60}{\Delta t\cdot\rho_{myo}} (5)$$

where $\rho_{myo}$ is the myocardial density. Clinical $C_{aif}\left( t \right)$ and $C_{myo}\left( t \right)$ curves represent the concentration in blood plasma where CA is diffusible, therefore MBF for the whole blood can be obtained by correcting with an additional factor for the myocardial blood hematocrit fraction [9]. For the analysis of clinical data in this study, a myocardial density of 1.05 g/mL and a hematocrit of tissue blood of 0.42 were used [8,9].

The least-squares solution to equation (4) is affected by noise in the input data that results in unwanted oscillations in $R_{F}\left( t \right)$, rendering the minimization problem ill-posed. This is particularly important in clinical perfusion imaging where the signal-to-noise ratio (SNR) at the pixel level typically varies between 10-30 [10-12]. Therefore, different methods can be employed to stabilize the solution and reliably quantify $R_{F}\left( t \right)$ and MBF.

**Fermi function-constrained deconvolution**

One method to stabilize the solution is to constrain the shape of $R_{F}\left( t \right)$ to a specific analytical function. The most popular function to model $R_{F}\left( t \right)$ is the Fermi function [3,13]:

$$R_{F}\left( t \right)=\frac{A}{e^{\frac{t-\mu}{k}}+1} (6)$$

MBF is measured from $R_{F}\left( t \right)$ providing the best fit to the data during least-squares minimization of equation (4). The three free parameters, $A$, $\mu$ and $k$, do not have a direct physiological interpretation and the function does not model explicitly the prolonged clearance of CA from the myocardial tissue, therefore it generally describes a single, well-mixed vascular compartment [3]. Different formulations of the Fermi function have been used over time, which have a similar or identical performance [13-17]. Additionally, the function can be multiplied by a Heaviside step function to account for the time delay due to tracer arrival in the myocardial tissue, which may be included as a free parameter in the fitting process or explicitly estimated in a prior step [13,18]; the latter was used in this study.

For simulations performed here, only parameter $k$ was varied, describing the rate by which the Fermi function decays, while the other two parameters were kept fixed at $A=1$ and $\mu=0$; the latter ensures no simulation of an initial plateau before CA arrival, permitting reliable analysis of the data with the one-compartment model (equation (8) below). All parameter values used lie within the typical range measured in clinical data using Fermi function-constrained deconvolution.

**Truncated singular value decomposition**

One can also perform non-parametric deconvolution without a priori knowledge of the shape of $R_{F}\left( t \right)$ using model-independent algebraic techniques. A common technique employs singular value decomposition (SVD) [19]. This constructs matrices $U, \Sigma$ and $V^{T}$ so that

$$\mathbf{A}\boldsymbol{=}U\Sigma V^{T}=\sum_{i=1}^{n} u_{i}\sigma_{i}v_{i}^{T}$$

where $\Sigma=(\sigma_{1},\ldots,\sigma_{n})$ is a diagonal matrix of the same size as $\mathbf{A}$ and contains its singular values in decreasing order, while $U=(u_{1},\ldots,u_{n})$ and $V=(v_{1},\ldots,v_{n})$ are both square orthogonal matrices ($U^{T}U=V^{T}V=I$). The simplest method to regularize SVD is by discarding elements in $\mathbf{A}$ that induce undesired oscillations to the solution, a process called truncated SVD (TSVD). The number of included singular values is determined by a chosen truncation parameter $k$, such that the solution is

$$R_{k}=\sum_{i=1}^{k} \frac{u_{i}^{T}\mathbf{M}}{\sigma_{i}}v_{i} (7)$$

Note that if $k=n$ then the naive solution to equation (3) is obtained. TSVD favors cases with high SNR and long MTT, otherwise severe misestimation of MBF can occur due to oscillations in $R_{F}\left( t \right)$ that may smooth its peak.

A more gradual solution can be obtained with the use of Tikhonov regularization. This expects a nonzero residual norm $\left\| \mathbf{AR-M} \right\|^{2}$ and introduces a regularization term to obtain a smaller residual norm relative to the solution norm, $\left\| L\mathbf{R} \right\|^{2}$. Equation (4) is then reformulated to

$$\hat{\mathbf{R}}=arg min\left\{ \left\| \mathbf{AR-M} \right\|^{2}\boldsymbol{+}\lambda^{2}\left\| L\mathbf{R} \right\|^{2} \right\}$$

$L$ is a matrix operator of order $n$ with full row rank, chosen based on a priori information, while $\lambda$ is a regularization parameter determining how much weight is given to minimization of the solution norm relative to the residual norm. In the classical version of Tikhonov regularization, $L$ is the identity matrix $I$, in which case the SVD solution (equation (7)) can be written as [20]

$$R_{\lambda}=\sum_{i=1}^{n} \frac{\sigma_{i}^{2}}{\sigma_{i}^{2}+\lambda^{2}}\frac{u_{i}^{T}\mathbf{M}}{\sigma_{i}}v_{i}=\sum_{i=1}^{n} \frac{\sigma_{i}}{\sigma_{i}^{2}+\lambda^{2}}u_{i}^{T}\mathbf{M}v_{i}$$

The first SVD components, corresponding to singular values greater than $\lambda$, contribute the most to the solution, whereas the last SVD components are damped considerably.

In perfusion quantification, the truncation parameter $k$ is often chosen by setting a cut-off level on the first singular value $\sigma_{1}$, so that all singular values larger than this are preserved [21,22]. The regularization parameter $\lambda$ can be estimated by means of the *L*-curve criterion [23]. In this study, the truncation parameter $k$ was chosen at 15% of the first singular value, which is a suitable cut-off level for the noise levels used in the simulated data [21,22]. A single fixed regularization parameter $\lambda$ was estimated based on a subset of the data by means of the *L*-curve criterion [21].

**One-compartment modelling**

Apart from methods that stabilize the deconvolution process, tracer-kinetic modeling relies on the compartmentalization of tissue and mathematical modeling of the exchange of CA between compartments. One-compartment models assume that the permeability between the vascular and extracellular compartments is sufficiently high that the two can be considered as a single compartment. A simple one-compartment model where the outflow of blood is proportional to the amount of CA inside it can be defined as

$$C_{myo}\left( t \right)=C_{aif}\left( t \right)Fe^{-\frac{F}{\nu_{p}}t} (8)$$

where $\nu_{p}$ is the fraction of distribution volume within the myocardium [24]. Parameter $F$ provides an estimate of MBF after correction for the time scale as described above. The equation can be solved as a nonlinear least-squares problem that does not require estimation of an impulse response. Here, the trust-region-reflective algorithm was used, with bound constraints on the free parameters [25].

**References**

1. Zierler KL. Theoretical Basis of Indicator-Dilution Methods For Measuring Flow and Volume. Circ Res. 1962;10:393-407.

2. Zierler KL. THEORY OF THE USE OF ARTERIOVENOUS CONCENTRATION DIFFERENCES FOR MEASURING METABOLISM IN STEADY AND NON-STEADY STATES. J Clin Invest. 1961;40:2111-2125.

3. Jerosch-Herold M. Quantification of myocardial perfusion by cardiovascular magnetic resonance. J Cardiovasc Magn Reson. 2010;12:57.

4. Sourbron S, Luypaert R, Morhard D, Seelos K, Reiser M, Peller M. Deconvolution of bolus-tracking data: a comparison of discretization methods. Phys Med Biol. 2007;52:6761-78.

5. Sourbron SP, Buckley DL. Classic models for dynamic contrast-enhanced MRI. NMR Biomed. 2013;26:1004-27.

6. Jerosch-Herold M, Wilke N, Wang Y, Gong GR, Mansoor AM, Huang H et al. Direct comparison of an intravascular and an extracellular contrast agent for quantification of myocardial perfusion. Cardiac MRI Group. Int J Card Imaging. 1999;15:453-64.

7. Pack NA, DiBella EV, Rust TC, Kadrmas DJ, McGann CJ, Butterfield R et al. Estimating myocardial perfusion from dynamic contrast-enhanced CMR with a model-independent deconvolution method. J Cardiovasc Magn Reson. 2008;10:52.

8. Zarinabad N, Chiribiri A, Hautvast GL, Ishida M, Schuster A, Cvetkovic Z et al. Voxel-wise quantification of myocardial perfusion by cardiac magnetic resonance. Feasibility and methods comparison. Magn Reson Med. 2012;68:1994-2004.

9. Kunze KP, Nekolla SG, Rischpler C, Zhang SH, Hayes C, Langwieser N et al. Myocardial perfusion quantification using simultaneously acquired (13) NH3 -ammonia PET and dynamic contrast-enhanced MRI in patients at rest and stress. Magn Reson Med. 2018;80:2641-2654.

10. Broadbent DA, Biglands JD, Ripley DP, Higgins DM, Greenwood JP, Plein S et al. Sensitivity of quantitative myocardial dynamic contrast-enhanced MRI to saturation pulse efficiency, noise and t1 measurement error: Comparison of nonlinearity correction methods. Magn Reson Med. 2016;75:1290-300.

11. Hunold P, Maderwald S, Eggebrecht H, Vogt FM, Barkhausen J. Steady-state free precession sequences in myocardial first-pass perfusion MR imaging: comparison with TurboFLASH imaging. Eur Radiol. 2004;14:409-16.

12. Fenchel M, Helber U, Simonetti OP, Stauder NI, Kramer U, Nguyen CN et al. Multislice first-pass myocardial perfusion imaging: Comparison of saturation recovery (SR)-TrueFISP-two-dimensional (2D) and SR-TurboFLASH-2D pulse sequences. J Magn Reson Imaging. 2004;19:555-63.

13. Jerosch-Herold M, Wilke N, Stillman AE. Magnetic resonance quantification of the myocardial perfusion reserve with a Fermi function model for constrained deconvolution. Med Phys. 1998;25:73-84.

14. Biglands J, Magee D, Boyle R, Larghat A, Plein S, Radjenovic A. Evaluation of the effect of myocardial segmentation errors on myocardial blood flow estimates from DCE-MRI. Phys Med Biol. 2011;56:2423-43.

15. Sourbron S. Technical aspects of MR perfusion. Eur J Radiol. 2010;76:304-13.

16. Papanastasiou G, Williams MC, Kershaw LE, Dweck MR, Alam S, Mirsadraee S et al. Measurement of myocardial blood flow by cardiovascular magnetic resonance perfusion: comparison of distributed parameter and Fermi models with single and dual bolus. J Cardiovasc Magn Reson. 2015;17:17.

17. Hsu LY, Rhoads KL, Holly JE, Kellman P, Aletras AH, Arai AE. Quantitative myocardial perfusion analysis with a dual-bolus contrast-enhanced first-pass MRI technique in humans. J Magn Reson Imaging. 2006;23:315-22.

18. Zarinabad N, Hautvast G, Sammut E, Arujuna A, Breeuwer M, Nagel E et al. Effects of tracer arrival time on the accuracy of high-resolution (voxel-wise) myocardial perfusion maps from contrast-enhanced first-pass perfusion magnetic resonance. IEEE Trans Biomed Eng. 2014;61:2499-2506.

19. Ostergaard L, Weisskoff RM, Chesler DA, Gyldensted C, Rosen BR. High resolution measurement of cerebral blood flow using intravascular tracer bolus passages. Part I: Mathematical approach and statistical analysis. Magn Reson Med. 1996;36:715-25.

20. Hansen PC. Deconvolution and regularization with Toeplitz matrices. Numer Algorithms. 2002;29:323-378.

21. Ostergaard L, Sorensen AG, Kwong KK, Weisskoff RM, Gyldensted C, Rosen BR. High resolution measurement of cerebral blood flow using intravascular tracer bolus passages. Part II: Experimental comparison and preliminary results. Magn Reson Med. 1996;36:726-36.

22. Meijs M, Christensen S, Lansberg MG, Albers GW, Calamante F. Analysis of perfusion MRI in stroke: To deconvolve, or not to deconvolve. Magn Reson Med. 2016;76:1282-90.

23. Hansen PC. Analysis of Discrete Ill-Posed Problems by Means of the L-Curve. Siam Rev. 1992;34:561-580.

24. Vallee JP, Sostman HD, MacFall JR, Wheeler T, Hedlund LW, Spritzer CE et al. MRI quantitative myocardial perfusion with compartmental analysis: a rest and stress study. Magn Reson Med. 1997;38:981-9.

25. Kunze KP, Rischpler C, Hayes C, Ibrahim T, Laugwitz KL, Haase A et al. Measurement of extracellular volume and transit time heterogeneity using contrast-enhanced myocardial perfusion MRI in patients after acute myocardial infarction. Magn Reson Med. 2017;77:2320-2330.

**
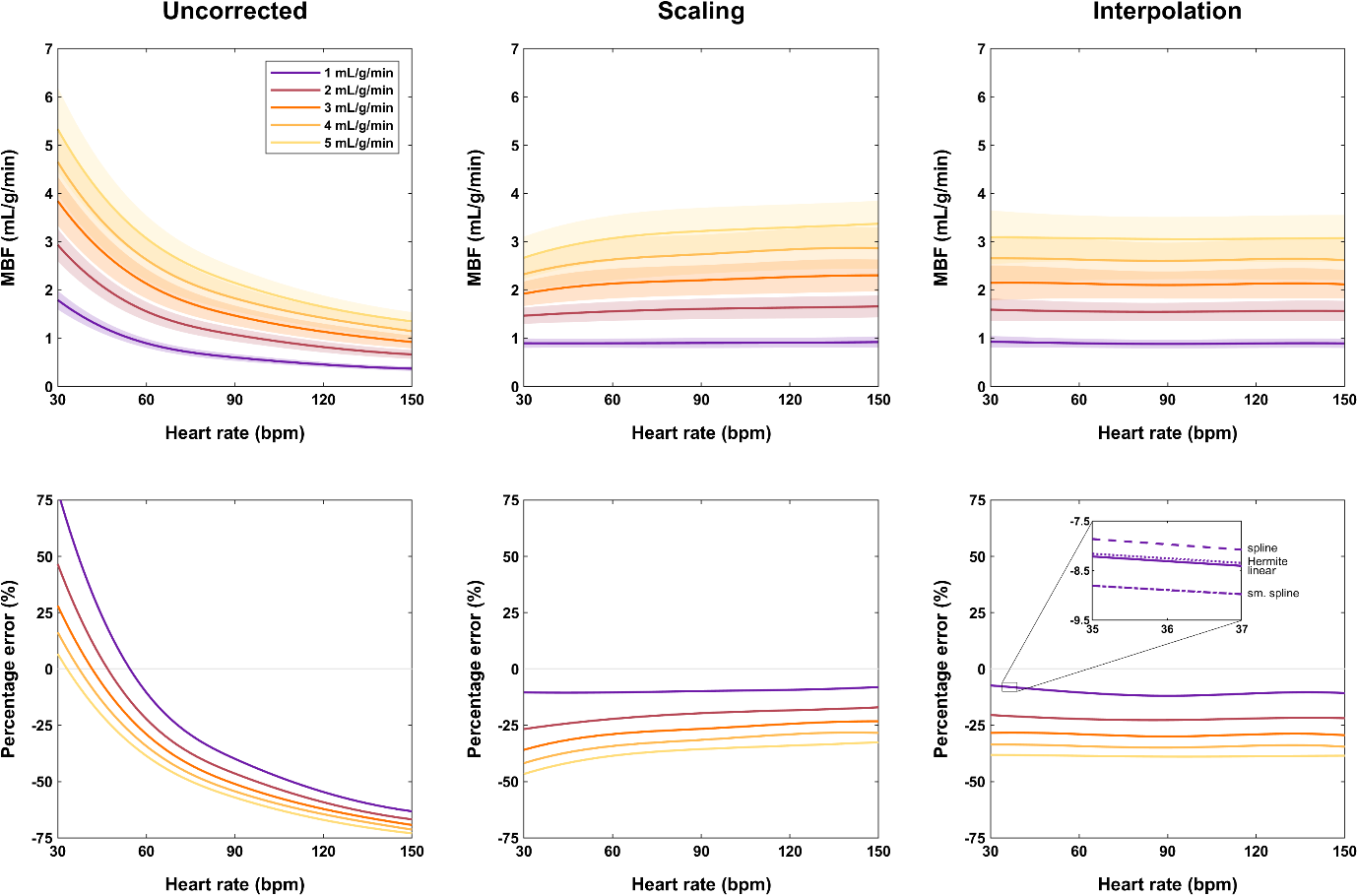
**

**Supporting Information Figure S1:** Estimated myocardial blood flow in simulated data for truncated singular value decomposition with Tikhonov regularization. Plots show blood flow (top row) and its percentage error (bottom row) for 3 approaches: no correction for temporal resolution (left column), MBF scaling correction (middle column), and data interpolation to 60 bpm (right column). 20 seconds were used for quantification. The lines indicate the mean estimate and the shaded areas the standard deviation (except for percentage error for clarity), with cubic spline fitting used to generate a continuous curve for each perfusion rate over all heart rates. The inset plot for interpolation shows the differences between interpolation algorithms for 1 mL/g/min reference blood flow.

**
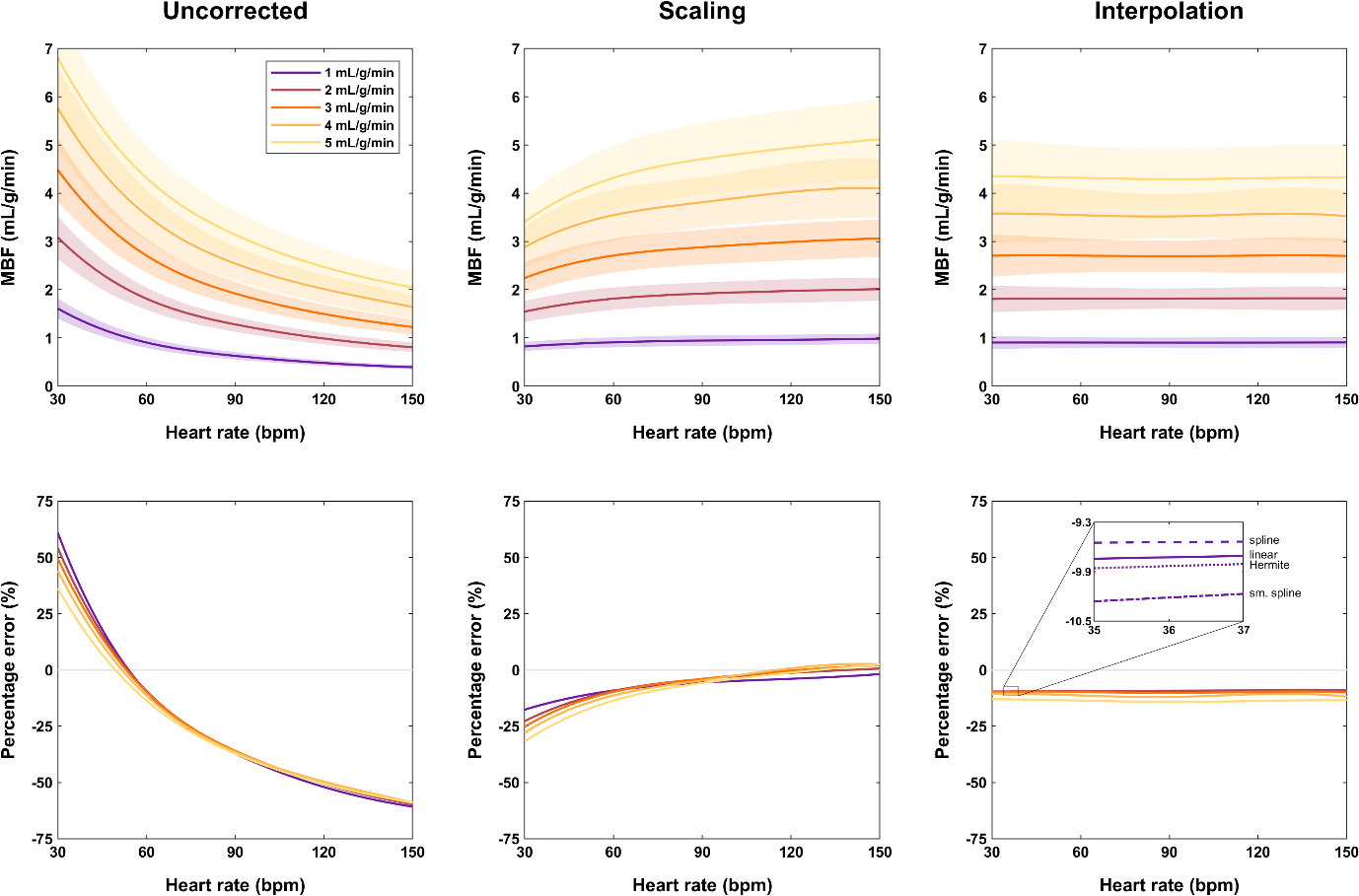
**

**Supporting Information Figure S2:** Estimated myocardial blood flow in simulated data for one-compartment modeling. Plots show blood flow (top row) and its percentage error (bottom row) for 3 approaches: no correction for temporal resolution (left column), MBF scaling correction (middle column), and data interpolation to 60 bpm (right column). 20 seconds were used for quantification. The lines indicate the mean estimate and the shaded areas the standard deviation (except for percentage error for clarity), with cubic spline fitting used to generate a continuous curve for each perfusion rate over all heart rates. The inset plot for interpolation shows the differences between interpolation algorithms for 1 mL/g/min reference blood flow.

**
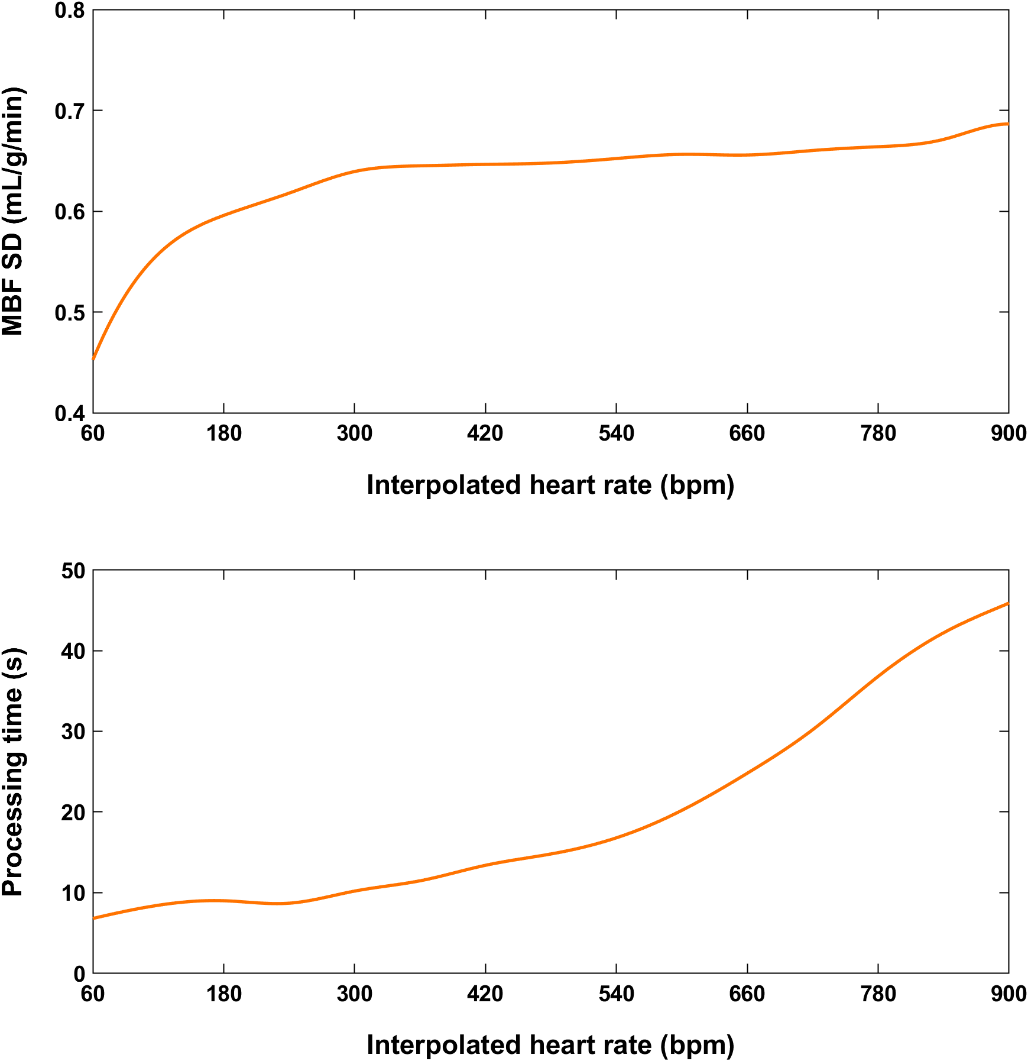
**

**Supporting Information Figure S3:** Precision and processing time in myocardial blood flow (MBF) quantification. Fermi function-constrained deconvolution was used, following cubic spline interpolation to various temporal resolutions. MBF precision, given as the standard deviation (SD) of MBF, was measured for an input heart rate of 90 bpm and a reference flow rate of 3 mL/g/min, which are typical values during hyperemia. The processing time was the mean time required to process 1000 pairs of curves at each interpolated heart rate (including interpolation and fitting), from all 25 combinations of input heart rates and reference flow rates. Cubic spline fitting was used to generate a continuous curve for each plot. Corresponding MBF percentage errors are shown in Figure 5 in the main manuscript.

**
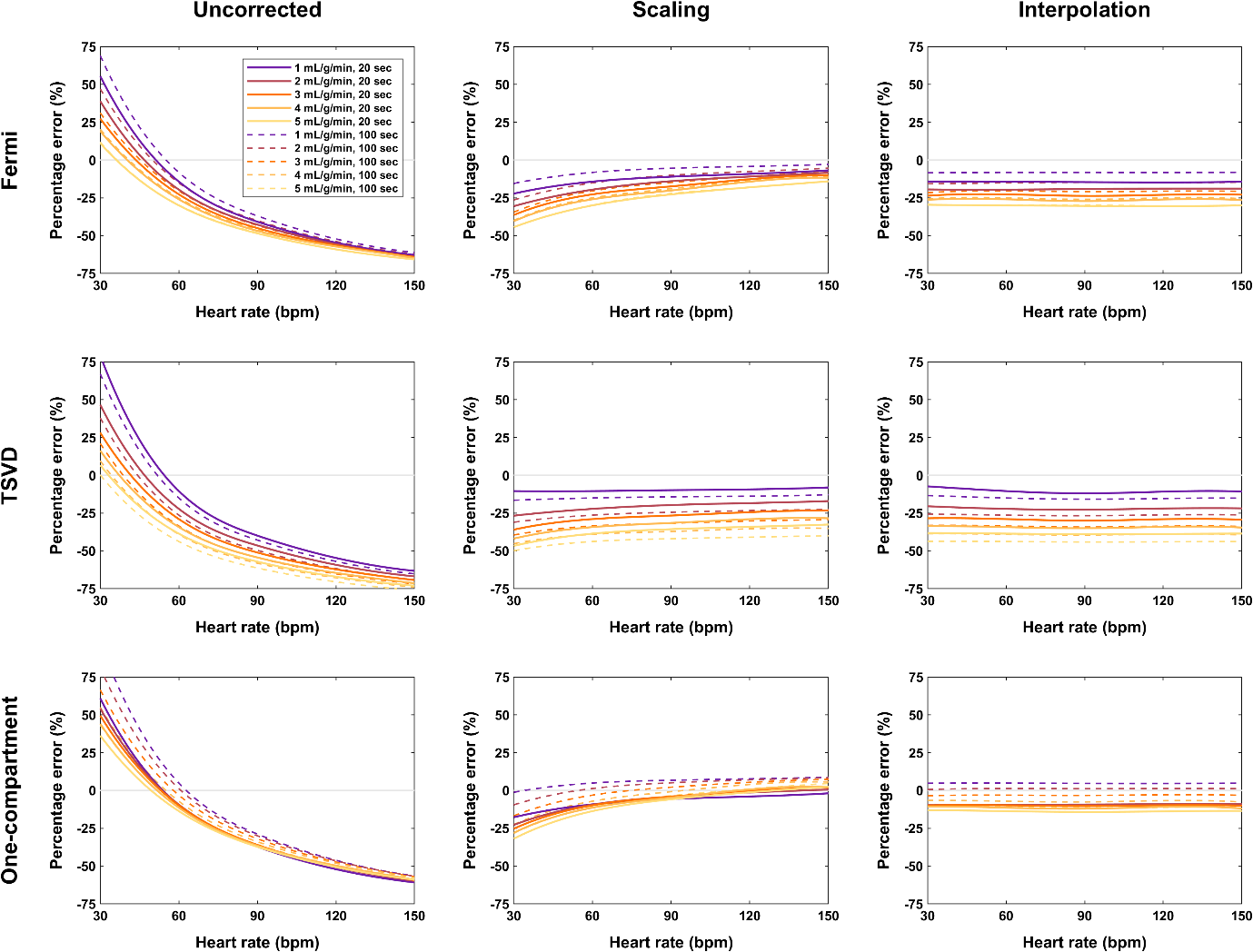
**

**Supporting Information Figure S4:** Percentage error in myocardial blood flow in simulated data for 20 and 100 seconds temporal window for quantification. Each rows shows the error for a different quantification method: Fermi function-constrained deconvolution (top row), truncated singular value decomposition with Tikhonov regularization (TSVD; middle row) and one-compartment modeling (bottom row). Columns show the error without correction for temporal resolution (left column), for MBF scaling correction (middle column), and after data interpolation to 60 bpm (right column). The standard deviation is not shown for clarity. Cubic spline fitting was used to generate a continuous curve for each perfusion rate over all HR values. The extend and direction of errors differs across quantification methods but is largely independent of the temporal resolution of the data and methods for correction.


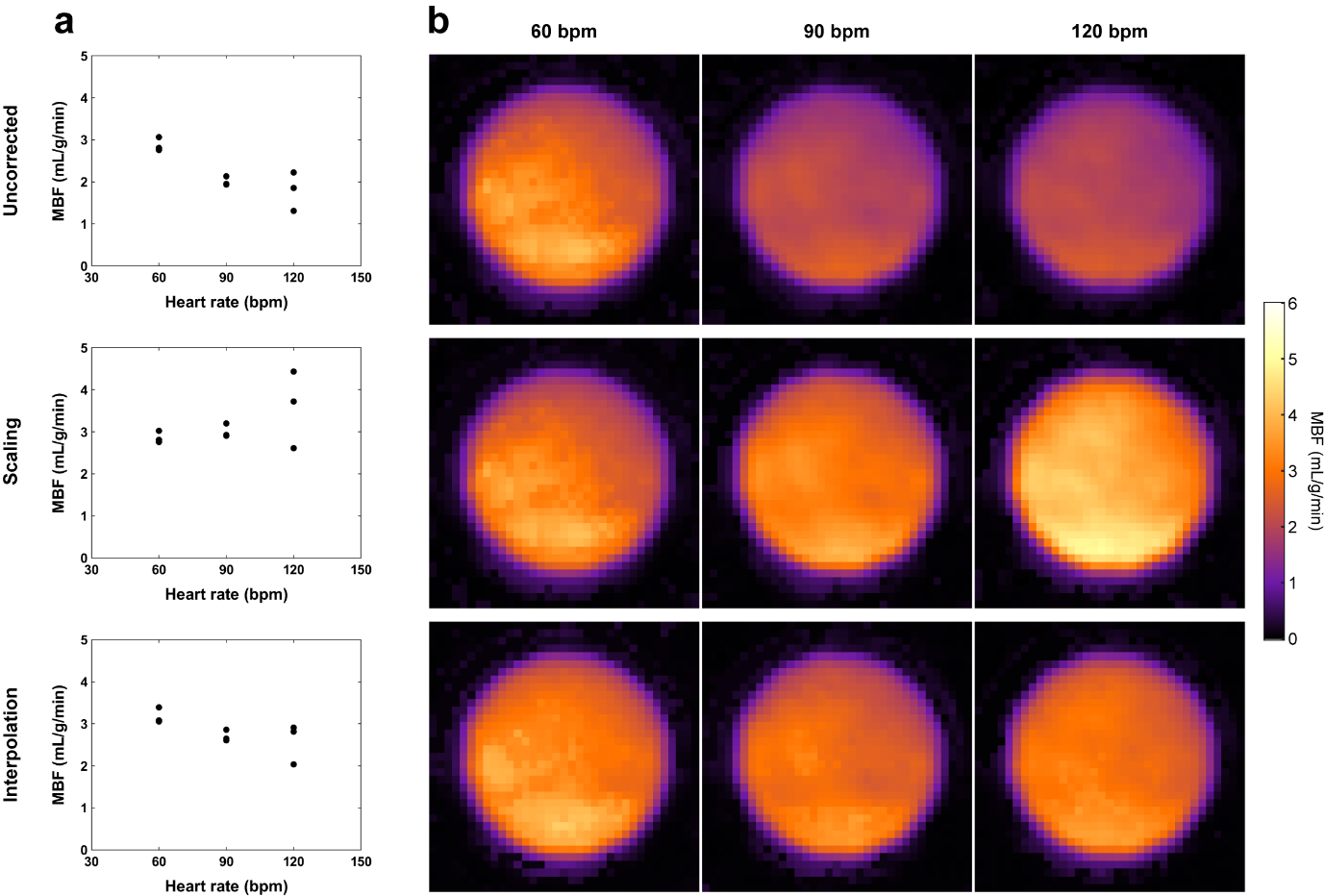


**Supporting Information Figure S5:** Effect of temporal resolution on pixel-wise myocardial blood flow (MBF) maps for the phantom. Three repeated scans for each of 3 different simulated heart rates were acquired, all for a fixed flow of 3 mL/g/min. Plots show the mean flow without correction for temporal resolution, for MBF scaling correction, and after data interpolation to 240 bpm (a). Representative pixel-wise maps are also shown (b). MBF scaling correction can produce outlying measurements and increase the variability in quantification, which is not the case with interpolation correction.


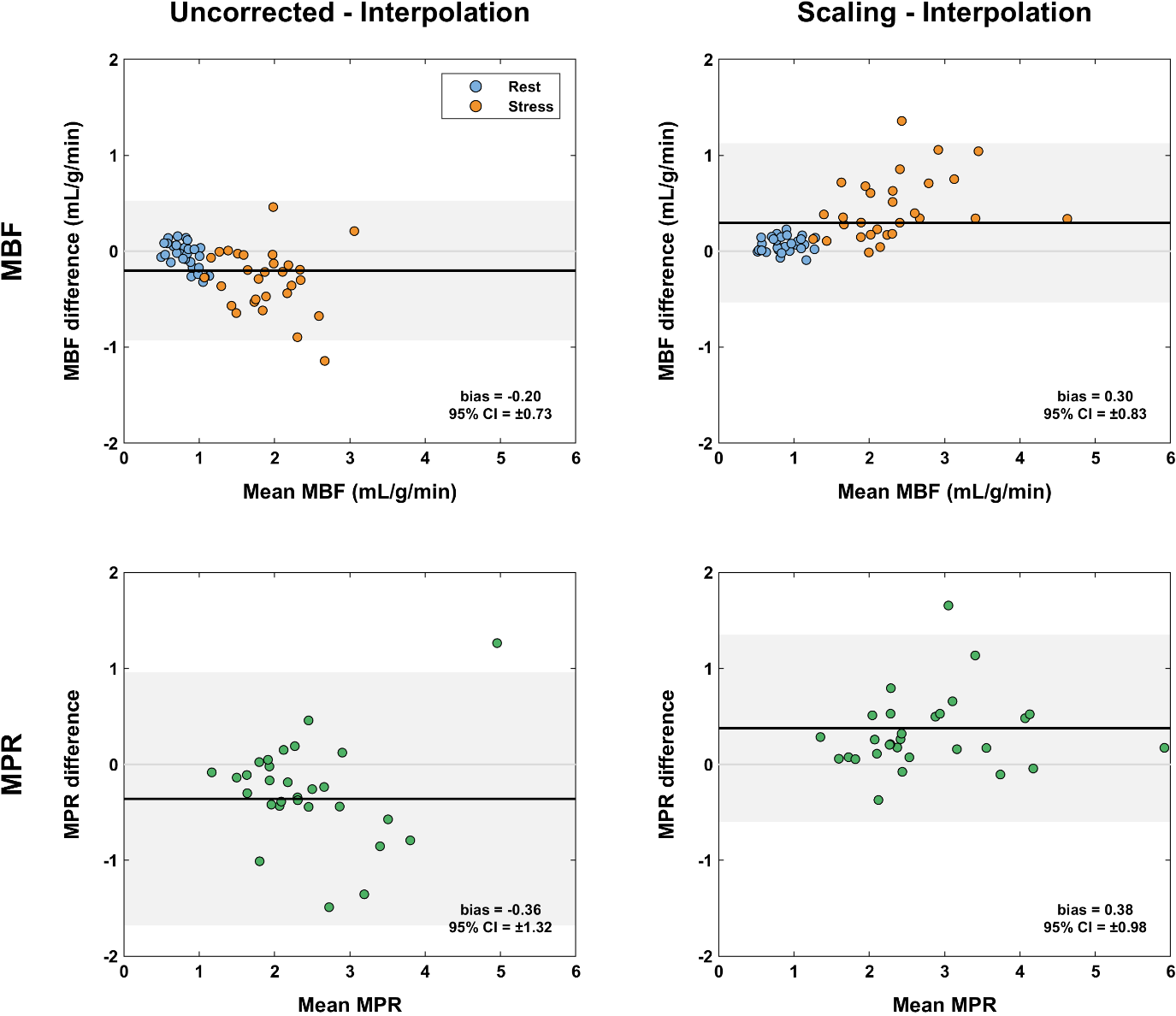


**Supporting Information Figure S6:** Bland-Altman plots for myocardial blood flow (MBF) and myocardial perfusion reserve (MPR). The most accurate approach based on the phantom study (quantification after data interpolation to 240 bpm) was compared with uncorrected quantification (left column) and quantification after scaling by the inverse of the sampling interval (right column). The shaded areas show the 95% confidence intervals (CI).
